# Long‐term culture of skin biopsies: maintenance of fibroblast production and competency of reprogramming

**DOI:** 10.1002/2211-5463.70136

**Published:** 2025-12-19

**Authors:** Sudiksha Rathan‐Kumar, Michael A. Ripperger, Grant M. Westlake, Kevin C. Ess

**Affiliations:** ^1^ Department of Pediatrics Vanderbilt University Medical Center Nashville TN USA; ^2^ Department of Pediatrics University of Colorado Anschutz Medical Campus Aurora CO USA; ^3^ Department of Cell and Developmental Biology Vanderbilt University Nashville TN USA; ^4^ Department of Biomedical Informatics Vanderbilt University Medical Center Nashville TN USA

**Keywords:** fibroblasts, reprogramming, skin culture

## Abstract

Primary fibroblasts are widely used in a variety of experimental and therapeutic studies. Patient‐derived skin biopsies are an accessible way to generate dermal fibroblasts for wound and burn therapeutics and can be easily reprogrammed to induced pluripotent stem cells (iPSCs). Despite the increasing use and interest in skin biopsies, there is limited information regarding the culturing potential of a single biopsy and the effects of extended culture on fibroblast formation and reprogramming potential. To better understand the potential of long‐term skin biopsy culture, we cultured biopsy samples for 6–16 months, resulting in 6–16 generations of explant reculturing and then analyzed subsequent generations of fibroblasts. Our results showed that fibroblast morphology and physiology are maintained over time, but although older generations remained proliferative, they did so at a decreased rate. Gene expression analyses uncovered transcriptional changes with long‐term skin culture, but deep DNA sequencing did not reveal any large deletions or amplifications. Spontaneous DNA mutations in fibroblast generations appeared to be random and not enriched for any specific signaling pathways. Importantly, fibroblasts generated after 16 months and over 16 generations in explant culture retained competency for reprogramming into induced pluripotent stem cells. Taken together, our results support long‐term culture of skin biopsies to generate large numbers of primary fibroblasts. These cells maintain their identity and integrity, enabling the study of fibroblast maintenance as well as rare human disorders.

## Graphical abstract

Skin biopsies taken from a patient with an ultra‐rare disorder as well as controls were cultured for up to 473 days. The chunks of skin were serially transferred to a new culture plate when confluent with fibroblasts. Different generations of fibroblasts were analyzed for cell and molecular properties, proliferation, and competence for reprogramming to induced pluripotent stem cells.

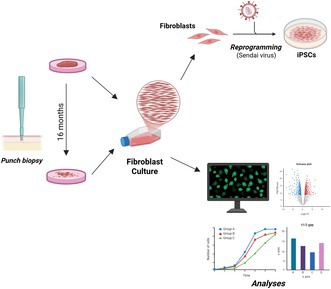

**Abbreviations**
CC3: Cleaved Caspase 3
CNV: copy number variations
ECM: extracellular matrix
FGF: fibroblast growth factor
FSP1: fibroblast‐specific protein 1
Gen1: first generation
GEO: Gene Expression Omnibus
GOEA: Gene Ontology Enrichment Analysis
HAL: Hikeshi Associated Leukodystrophy
H&E: hematoxylin and eosin
iPSCs: induced pluripotent stem cells
PFA: paraformaldehyde

Fibroblasts are one of the most abundant cells in the human body. First described in 1858, this cell type is present in a variety of organs including the skin, lungs, heart, and skeletal musculature [1, 2]. Due to their tissue‐specific functions, they originate from various lineages, converging onto a fibroblast fate. They have been best studied in the skin, where dermal fibroblasts form a strong foundation through deposition of extracellular matrix, supporting keratinocytes, sweat glands, and hair follicles [1]. Fibroblast progenitors are of mesenchymal origin and generate dermal fibroblasts as well as dermal adipocytes [3].

Dermal fibroblasts play an important role in wound healing, initially interacting with immune cells during the inflammatory phase and improving the local immune response [4, 5]. Fibroblasts also have a primary role during proliferative and skin remodeling, contributing to angiogenesis and secreting extracellular matrix (ECM) molecules [6, 7]. They also differentiate into myofibroblasts, which regulate wound contraction and tissue remodeling to complete the wound healing process [8, 9].

Due to this function, fibroblasts have also been highly studied regarding wound recovery and burn therapeutics [10]. Studies and clinical trials have shown that the application of fibroblast growth factor (FGF) improves wound healing time and scar appearance [11]. Recent studies in animal models have demonstrated that the transplantation of both allogeneic and autogenic fibroblasts onto wounds and ulcers improved wound healing [12, 13, 14, 15, 16]. Further studies utilized microcarriers and engineered sheets containing fibroblasts and other factors to promote wound healing [17, 18, 19]. Fibroblasts have also been used in skin replacement treatments for burn patients, with autologous cells being cultivated and transplanted onto the patient, leading to fewer complications during healing [20, 21]. Therefore, cultured fibroblasts have various applications in burn and wound healing therapeutics; however, there are limitations due to the required quantity and lifespan of the cells, with long‐term culture resulting in cellular senescence [15, 22, 23].

Another vital utilization of fibroblasts is their ability to be reprogrammed to induced pluripotent stem cells (iPSCs). Skin punch biopsies can be readily performed on healthy volunteers as well as individuals with diseases, and primary fibroblast cell lines established [24, 25, 26]. Primary fibroblasts can be reprogrammed to iPSCs by exogenous expression of ‘Yamanaka factors’ *SOX2*, *cMYC*, *KLF4*, and *OCT3/4* utilizing episomal vectors or non‐integrative Sendai virus transduction [27, 28, 29, 30]. A major application of this approach is to model rare genetic disorders, as fibroblasts retain the germline mutation of each patient, and after generation of iPSCs, can be differentiated to various lineages of interest such as neurons, hepatocytes, or cardiomyocytes [31, 32].

Human‐derived primary cells have then greatly expanded the field of biomedical research. In rare disease research, human‐derived cell models have proven invaluable in understanding disease mechanisms and developing therapeutics [33, 34]. Governmental policies have also increased interest in the use of iPSCs for drug discovery and as part of clinical trials [35, 36]. However, for extremely rare diseases, due to the low number of patients, acquiring such cells has been a challenge for researchers and clinicians. Furthermore, the limiting effects of disease symptoms and the possible financial and medical burdens of travel might prevent participation and contribution of biopsy samples from certain patients and regions. Therefore, any skin biopsy and the resulting fibroblasts are invaluable tools, especially for rare disorders.

Previous research has demonstrated the applicability to cryopreserve biopsies for later culture [37]. However, there is minimal investigation on the culturing potential of a single biopsy and the effects of extended culture on fibroblast formation and reprogramming potential. ‘Classical’ papers from the 1960s and 1970s showed the ability for long‐term fibroblast culture but were limited to 6 months or less [38]. The demonstration of the Hayflick limit used long‐term cell culture but focused on individual cell doublings, finding that normal human diploid cells can generally divide approximately 50 times before senescence [39]. Other more recent methods have been used to improve long‐term culture of human fibroblasts by utilizing cultured dermis and spheroids for up to 6 months [40].

We now report a long‐term study of biopsy‐derived fibroblast cultures, including the retained ability to reprogram to iPSCs from long‐term fibroblast cultures. Our results indicate that patient‐derived biopsies can produce very large numbers of fibroblasts for extended periods of time, and these fibroblasts from long‐term culture maintain genomic and physiological features. Our results address the ability of skin explant cultures to produce large quantities of fibroblasts and retain the potential to become patient‐derived iPSCs. These results have broad applications in wound therapeutics and fibroblast physiology.

## Materials and methods

### Skin biopsies

All skin biopsies were conducted under the approval of Health Sciences Review Committee #2 of the Institutional Review Board of Vanderbilt University Medical Center, IRB #080369. All research was performed in accordance with relevant guidelines/regulations including those in accordance with the Declaration of Helsinki. Written informed consent/assent was obtained from each subject, and a single 3 mm punch skin biopsy was obtained from the posterior aspect of an arm of each subject. Tissue was dissected in half longitudinally, cut downwards from the exterior surface. Each half was then plated in a single well of a 6‐well culture plate. A sterile glass slide cover was placed on the skin section. 3 mL of DMEM Complete [1× DMEM (Gibco 11995‐065, Grand Island, NY, USA), 10% FBS (Corning 35‐015‐CV, Manassas, VA, USA), 100 U·mL^−1^ Penicillin/Streptomycin (Gibco 15140‐122), 1× MEM Non‐Essential Amino Acids (Gibco 11140‐050)] was added to each well, completely submerging the biopsy and slide. Plates were incubated at 37 °C and 5% CO_2_, gently aspirating and changing media twice a week. After 1–3 weeks, fibroblast‐appearing cells began to migrate out from the skin chunk. When confluent, the still intact skin chunks were moved to a new 6‐well plate, with a fresh sterile glass slide cover to keep the tissue adherent to the culture dish. Fibroblasts from the original well were passaged using 0.1% Trypsin–EDTA and plated on a 10 cm petri dish with DMEM Complete for further growth and expansion before cryopreservation.

For storage, cells were trypsinized and resuspended in fibroblast freezing media [50% DMEM Complete, 40% FBS (Corning 35‐015‐CV), 10% DMSO (Sigma‐Aldrich D8418, Saint Louis, MO, USA)]. The cell solution was then aliquoted into multiple cryotubes for each generation, frozen overnight at −80 °C, and then moved to liquid nitrogen for long‐term storage. Initial fibroblasts grown from the skin explant were denoted as the first generation (Gen1) and subsequent passaging and resulting fibroblast cultures as Gen2 through Gen16. HIKHOM‐1 was harvested at Gen16 after 16 months in culture; HIKHet‐2 was harvested at Gen6 after 6 months in culture; unrelated controls CH1‐CON and CH2‐CON were harvested at Gen1 after 1 month in culture.

### Patient selection

We focused our long‐term efforts on a punch biopsy from a male patient with Hikeshi Associated Leukodystrophy (HAL), a devastating autosomal recessive neurological disorder caused by homozygous loss of function mutations in the *HIKESHI* gene [41, 42, 43]. We also utilized cells obtained from healthy, asymptomatic first‐degree relatives who had heterozygous *HIKESHI* mutations as controls. We also obtained skin biopsies from two unrelated healthy individuals (CH1‐CON and CH2‐CON). The genotype of all lines was confirmed by DNA sequencing of the *HIKESHI* gene and further validated by immunostaining for HIKESHI protein.

### Immunohistochemistry

Skin biopsies were removed from the culture plate and fixed in 4% paraformaldehyde (PFA) for a minimum of 24 h. The biopsies were then transferred into 70% ethanol and submitted to the Vanderbilt Translational Pathology Shared Resource. Fixed tissue was embedded in paraffin and cut into 5 μm thick sections. One set of slides was stained with hematoxylin and eosin (H&E) [44]. The H&E‐stained slides were submitted to the Vanderbilt Digital Histology Shared Resource and imaged with the Leica SCN400 Slide Scanner (Leica Biosystems, Nussloch, Germany) at 40× magnification.

### Paraffin‐embedded slide immunofluorescence

Tissue sections were deparaffinized and rehydrated by immersion in xylene and a series of ethanol dilutions. Antigen retrieval was performed utilizing heated citrate buffer pH 6 [diH_2_O, 11.3 mm Trisodium citrate, 0.05% Tween 20]. Slides were incubated with blocking buffer [1× PBS, 5% Normal Donkey Serum (Sigma‐Aldrich D9663), 0.3% Triton X‐100] for 1 h at room temperature. Primary antibodies (Table 1) were diluted in the blocking buffer, and 100 μL was added to each well in a humidified chamber at 4 °C overnight. Secondary antibodies were diluted in blocking buffer and incubated at room temperature for 2 h. Slides were then washed twice with 1× PBS and mounted using Prolong Antifade DAPI mounting medium (Invitrogen P36935, Carlsbad, CA, USA) and allowed to dry overnight before imaging on the EVOS FL (Waltham, MA, USA) Auto microscope.

**Table 1:** Primary antibodies used, source, and dilution.

Antibody	Antibody registry ID	Usage
CC3	AB_2341188	1 : 500
FSP	AB_10000870	1 : 500
GATA4	AB_3105880	1 : 100
Ki67	AB_302459	1 : 250
Ki67	AB_10854564	1 : 400
PCNA	AB_2160343	1 : 500
Nanog	AB_10559205	1 : 200
Oct4	AB_823583	1 : 200
Smooth Muscle Actin	AB_262054	1 : 200
Sox1	AB_2809724	1 : 100
SSEA3	AB_177628	1 : 100
SSEA4	AB_528477	1 : 200
TRA‐1‐60	AB_2119183	1 : 200
β‐III‐Tubulin	AB_1904176	1 : 200
Vimentin	AB_10695459	1 : 500

### Chambered slide immunofluorescence

Eight‐well chambered slides (Ibidi 80841, Gräfelfing, Germany) were coated with 0.1 mg·mL^−1^ Poly‐l‐lysine (Sigma‐Aldrich P4707) for 20 min. Poly‐l‐lysine was aspirated, and slides were washed twice with dH_2_O and air dried for 2 h. Fibroblasts were harvested from a confluent T25 flask using 0.25% Trypsin–EDTA solution (Gibco 25200‐056) for 7 min at 37 °C before being spun down at 300 × ***g*** for 5 min, and the pellet was resuspended in 1 mL DMEM Complete. A Countess Cell Counter was used to count cells resuspended in DMEM Complete, plating 2000 cells into each well of the slide. Slides were incubated overnight at 37 °C. Media was aspirated, and slides were washed with 1× PBS before proceeding with antibody staining as above.

### Cell proliferation counting

Fibroblasts were plated on 8‐well chambered slides (Ibidi 80841) at 2500 cells per well. Cells were fixed and stained for expression of Ki67 and imaged on the EVOS FL Auto microscope at 10× magnification. evos Software captured 15 randomized images each from four wells. The captured images were analyzed and the total number of DAPI nuclei and Ki67 positive cells was counted. Cell counts were averaged for the well and each well was denoted as a data point for statistical analysis and graphing. Graphing was performed on graphpad prism.

### Proliferation assay

A proliferation assay was conducted on fibroblasts over a 7‐day period. Fibroblasts were harvested from a confluent T25 flask using 0.25% Trypsin–EDTA solution (Gibco 25200‐056) for 7 min at 37 °C. Fibroblasts were spun down, and the pellet was resuspended in 1 mL DMEM Complete. Cells were counted on the Nexcelom Cellometer Auto T4 Cell Counter and resuspended in DMEM Complete at 10 000 cells·mL^−1^. 100 μL of cell solution was added to 8 wells per genotype of 8 96‐well plates (1 for each day) for 1000 cells per well. Plates were incubated at 37 °C. A Thermofisher CyQuant Direct Cell Proliferation Assay (C35011) was utilized for analysis. For Day 0 to Day 7, the staining solution was prepared per the kit's instructions and 100 μL was added per well. Plates were incubated for 1 h at 37 °C. Plates were then read at 485/520 nm with a Biotek Synergy HT plate reader. Data were reported per previously published methods [45, 46, 47]. Data and statistics were compiled using biotek gen5 and graphpad prism.

### Wound healing assay

Fibroblasts were plated in a 24‐well plate at 100 000 cells per well with 4 wells for each of the 5 lines (HIKHet‐2 Gen1, Gen 6, and HIKHOM‐1 Gen1, Gen 6, Gen 16) [48]. Cells were incubated overnight at 37 °C, and the following day a scratch was made in the middle of the well using a sterile pipette tip. The cells were then imaged every 2 h for 24 h on the evos xl Core microscope at 4× magnification. The scratch area was quantified using the Wound Healing Size Tool PlugIn on imagej [49]. The data were analyzed, and the slope was determined to calculate the *t* ^1/2^ gap values across genotypes [50]. The statistics were compiled using graphpad prism.

### RNA sequencing

Fibroblasts were harvested and resuspended as above. RNA was extracted using the Qiagen RNAeasy Kit (Qiagen 74104, Hilden, Germany) and Qiagen RNA‐free DNase kit (Qiagen 79254). Samples were quantified using a NanoDrop and submitted to Vanderbilt Technologies for Advanced Genomics core laboratory.

Paired‐end reads were obtained from Illumina NovaSeq6000 PE150 Sequencing (San Diego, CA, USA). RNA sequence processing was performed with pyrpipe v0.0.5. First sequence adapters from the reads were trimmed with trimgalore v0.6.6 and aligned with star v2.7.7a to the Genome Reference Consortium Human Build 38 patch release 14 (GRCh38.p14). Assembly of alignments was done with StringTie v2.14 and the GRCh38.p14 NCBI RefSeq annotation assembly (GCF_000001405.40). Gene abundances were estimated using stringtie. Gene and transcript read counts were extracted for each sample with prepDE.py provided by Johns Hopkins University Centre for Computational Biology. pydeseq2 v0.3.5 was used to perform single and multiple factor differential expression analysis among Generations 1 and 6 for both cell lines and Generation 16 from HIKHOM‐1. Each paired read was processed independently.

Genes with adjusted *P*‐values by the Benjamini and Hochberg method under or equal to 0.001 and log fold change greater than 1 or less than −1 were selected for Gene Ontology Enrichment Analysis (GOEA) using goatools (v1.3.2) [51]. All human genes from Gencode annotations release 39 (GRCh38.p13) served as the background population set. Benjamini–Hochberg *P*‐values were obtained for each Gene Ontology biological process, cellular component, and molecular function pathway. Upregulated and downregulated genes were analyzed separately. Gene associations were taken from the human Gene Ontology 2023 library.

### Whole genome sequencing

Fibroblasts were harvested and resuspended as above. DNA was extracted using the DNAeasy Kit (Qiagen 69504). Samples were quantified using a NanoDrop (Wilmington, DE, USA). Samples were submitted to CD Genomics for low‐pass whole genome sequencing and analysis. Samples were run through a quality test and then utilized to create a construct library. The generated library was sequenced using the Illumina HiSeq sequencing platform, producing raw data. The raw data were processed to filter out various reads, including low‐quality and redundant reads. The sequencing quality score is determined, and the distribution of base content is calculated from the raw data. The reads were then aligned with a reference genome, and copy number variations (CNV) were determined. Deletions and duplications were identified and annotated with gene names or Ensembl IDs. Chromosome diagrams were generated for each genotype, depicting the CNVs. Statistics were conducted throughout the process for sequencing reads and alignment.

GOEA was performed in the same manner as with gene expression using DNA duplications and deletions from Generation 16 fibroblasts. Both duplications and deletions were considered a change to the gene and used as the study population. Changes already present in Generation 1 were removed from this set. A *P*‐value of 0.001 or less was used to identify modified pathways.

### Fibroblast reprogramming

Fibroblasts were reprogrammed using the CytoTune‐iPS 2.0 Reprogramming Kit (Invitrogen A16517). Fibroblasts were plated at 2 × 10^5^ cells per well of a Matrigel‐coated [Corning #354277, diluted in DMEM/F12 per manufacturer's instructions] 6‐well plate. Cells were fed with DMEM Complete. The following day (Day 0), 1 well was dissociated from the plate and the cell number was counted. The volume of virus required was calculated using the formula:

$$\mathrm{Vol}\;\text{of virus}\;\left(\mathrm{\mu L}\right)=\frac{\mathrm{MOI}\times \text{cells}\cdot {\mathrm{mL}}^{-1}}{\mathrm{CIU}\cdot {\mathrm{mL}}^{-1}\times 10^{-3}\mathrm{mL}\cdot {\mathrm{\mu L}}^{-1}}$$

Viruses hKOS and cMYC had a MOI of 5 and KLF4 a MOI of 3. Virus was added to DMEM complete without Penicillin/Streptomycin, adding 1 mL per well of cells. The following day (D1), the virus solution was carefully removed, and the cells were fed with DMEM Complete, changing the media every day from Day 1 to Day 6.

On Day 7, the cells were disassociated with 0.25% Trypsin and counted. They were plated on Matrigel‐coated 6‐well plates with densities of 5 × 10^4^, 7.5 × 10^4^ and 1 × 10^5^ cells per well. The cells were moved to Essential 8 Medium (Gibco A15169‐01) with Supplement (Gibco A15171‐01). Cells were then grown in E8 Medium, feeding every other day from Day 7 to Day 21. Cells were then moved to mTesR1 media (StemCell Technologies #85857, Vancouver, Canada). Individual colonies were identified and picked by scraping; colonies were transferred to a Matrigel‐coated 48‐well plate and grown on mTeSR1. Colonies were passaged over 5 generations and then fixed with 4% PFA for 30 min at room temperature. Immunofluorescence on colonies was done as above.

### Embryoid body trilineage assay

Reprogrammed iPSCs from HIKHet2 Gen1 and HIKHOM1 Gen16 were assessed using the trilineage embryoid body assay [52, 53]. iPSCs were grown to confluency, dissociated with ReLeSR (Stem Cell Technologies #100‐0483), and resuspended in mTeSR1 media. Cells were plated on low‐adherence plates for 4 days, feeding daily with a mixture of mTeSR1 and embryoid body media [1× DMEM/F12 + GlutaMAX (Gibco 10565‐018), 20% KnockOut Serum Replacement (Gibco 10828‐028), 100 U·mL^−1^ Penicillin/Streptomycin (Gibco 15140‐122), 1× MEM Non‐Essential Amino Acids (Gibco 11140‐050), 952.38 μm beta‐mercaptoethanol]. On Day 4, embryoid body clusters were plated on 3 Matrigel‐coated plates (one each for endoderm, mesoderm, and ectoderm). Cells were fed every day with embryoid body media. On Day 15, endoderm and mesoderm plates were fixed with 4% PFA for 20 min at room temperature. On Day 20, the ectoderm plate was fixed. All plates were stained using the immunofluorescence technique listed above.

### Statistical analysis

All analysis and graphs were produced using graphpad prism version 10.5.0 for Windows, GraphPad Software, Boston, MA, USA, www.graphpad.com. The conducted tests, number of replicates, and *P*‐values are listed in the corresponding Figure legend.

## Results

To generate primary dermal fibroblast cultures, we have been obtaining skin biopsies from patients with rare neurological disorders. The fibroblasts are used for direct functional experiments and for reprogramming to iPSCs. Given the very rare nature of some disorders we have studied, the initial skin biopsy from a patient with Hikeshi Associated Leukodystrophy (HAL, OMIM # 616881) was propagated further after initial fibroblasts were collected. We had previously observed the potential of human skin biopsies to continue to generate fibroblasts when the explant was placed in a new well. As patients with HAL can decompensate and even die following fever and infection, we strove to limit patient exposure to any invasive procedures including skin biopsy. We continued to propagate this individual chunk of skin for almost 16 months over 16 passages. As the fibroblasts were generated, the biopsy was replated for further growth, with each replating deemed a new ‘generation’. We noted the continued generation of fibroblasts, even from latter generations. We hypothesized that long‐term culture of the skin chunk did not impede fibroblast identity and function including cell morphology, gene expression, and ability to be reprogrammed to iPSCs. The HAL patient biopsy (HIKHOM‐1) was propagated for 16 generations (473 days in culture) with 6–10 tubes of fibroblasts frozen down at each passage upon transfer of the skin explant to a new well. Control cultures derived from healthy, non‐symptomatic first‐degree relatives with heterozygous *HIKESHI* mutations (HIKHet‐2) were also propagated for 6 generations, summarized in Table 2.

**Table 2:** Skin biopsies used and fibroblast generations post biopsy.

Label	Genotype	Generation	Days since biopsy
HIKHet‐2 Gen 1	HIKHet‐2	1	9 days
HIKHet‐2 Gen 6	HIKHet‐2	6	159 days
HIKHOM‐1 Gen 1	HIKHOM‐1	1	71 days
HIKHOM‐1 Gen 6	HIKHOM‐1	6	190 days
HIKHOM‐1 Gen 16	HIKHOM‐1	16	473 days

**Fig. 1:**
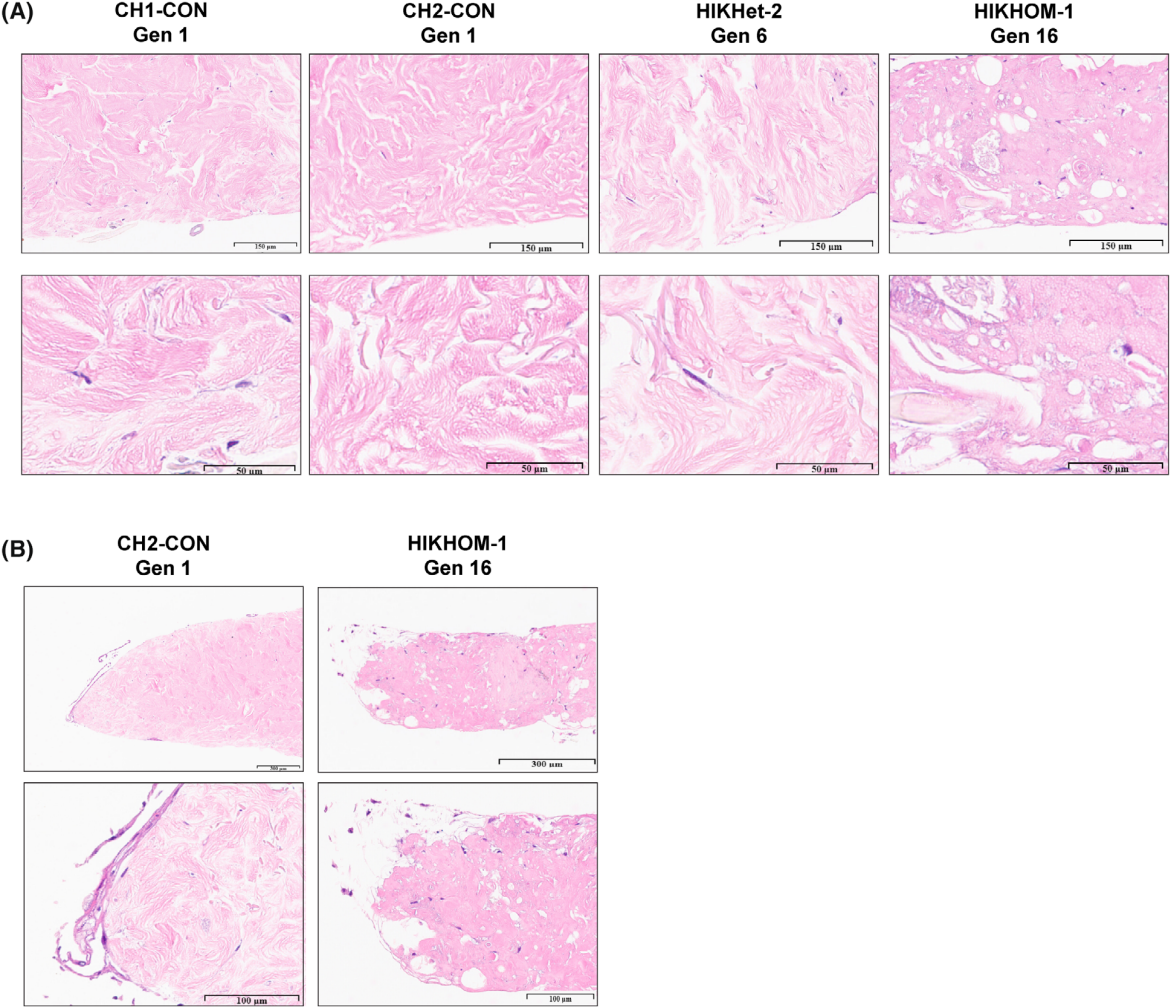
Skin biopsies display deterioration over time in culture. Skin biopsies from HIKHOM‐1, HIKHet‐2, and unrelated controls CH1‐CON and CH2‐CON were fixed, paraffin embedded, and H&E stained. Slides were imaged on an Aperio Versa 200 Bright Field microscope at 40× magnification. (A) As supported by vacuolar changes and tissue integrity, long‐term culture resulted in deterioration of the HIKHOM‐1 and HIKHet‐2 skin explants compared to the control pieces of skin. (B) HIKHOM‐1 displays loss of epidermis over culture compared to CH2‐CON. Representative images from each (*n* = 1) skin biopsy.

After final generation of fibroblasts, the HIKHOM‐1 and HIKHet‐2 skin explants were harvested, fixed, and sectioned. HIKHOM‐1 was harvested at Gen 16 and HIKHet‐2 was harvested at Gen 6. H&E stains of HIKHOM‐1 and HIKHet‐2 were compared to two unrelated control skin biopsies (CH1‐CON and CH2‐CON) harvested after one generation. HIKHOM‐1 skin biopsy appeared thinner and damaged compared to control skin biopsies. This could be potentially due to the long‐term culture over 16 months and multiple replating procedures over 16 generations. The HIKHet‐2 biopsy was also thinner and appeared more damaged than the controls (Fig. 1A). HIKHOM‐1 also displayed a lack of epidermis that is visible in the control, possibly due to degradation over culture (Fig. 1B).

**Fig. 2:**
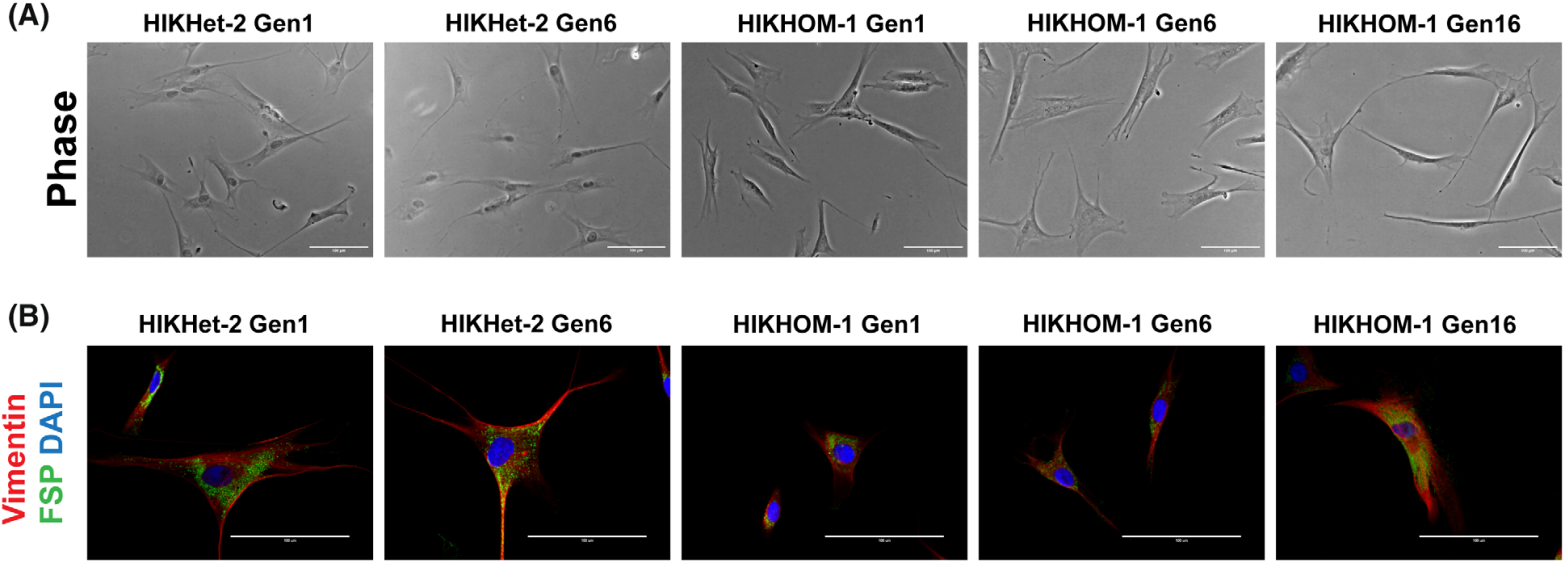
Fibroblasts retain spindle morphology and stain for markers. (A) Phase contrast imaging of fibroblasts displays characteristic spindle shape, indicating maintenance of morphology, scale bar: 100 μm. (B) Cells were fixed and stained for FSP and Vimentin, which were tested positive in all lines. Representative images from each (*n* = 1) skin biopsy. Scale bar: 100 μm.

All fibroblasts regardless of age, generation, and genotype exhibited the characteristic spindle morphology (Fig. 2), swirled patterns of growth, and contact inhibition when cultures were confluent [54]. The cells then seemed to retain fibroblast lineage and morphology over various generations, suggesting that long‐term biopsy culture does not affect cell type or morphology.

As we hypothesized that resident progenitor cells within each piece of skin remained proliferative and capable of producing fibroblasts, we used immunofluorescence for the expression of Fibroblast‐Specific Protein 1 (FSP1), a member of the S100 calcium‐binding protein superfamily [55]. FSP1 is present in fibroblasts and absent in epithelial, mesangial, and embryonic endoderm cells [56]. Cells were positive for FSP1 as well as the cytoskeletal protein Vimentin (Fig. 2), which has also been frequently used as a fibroblast marker [57, 58].

### Cell proliferation and death

**Fig. 3:**
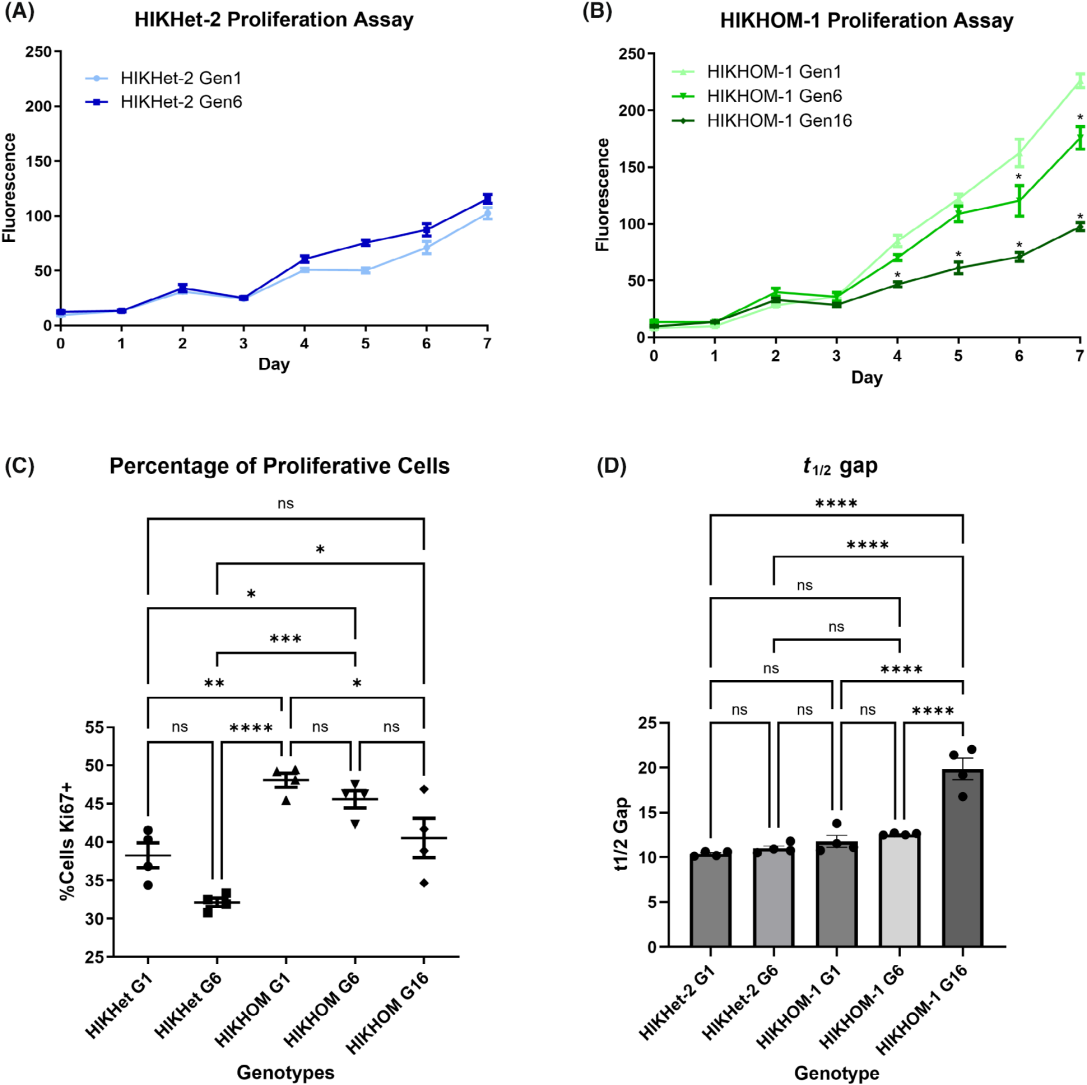
Later generations of fibroblasts remain proliferative but at a decreased rate. (A) Cells were plated and grown over 7 days. Cell numbers were measured each day utilizing the CyQuant Direct Cell Proliferation Assay. Analyses were conducted using repeated‐measures ANOVA for 8 replicate wells per day with a Bonferroni multiple comparison post‐test. HIKHet‐2 Gen 6 did not have any significant changes compared to HIKHet‐2 Gen 1 (Error bars = SEM). (B) Compared to HIKHOM‐1 Gen 1, HIKHOM‐1 Gen 6 and Gen 16 proliferation was significantly decreased by Day 6 and Day 4, respectively (Repeated‐measures ANOVA, Bonferroni post‐test, *n* = 8 wells per day per genotype, error bars = SEM, *P < 0.0001). (C) Fibroblasts were cultured, fixed, immunostained for Ki67, and imaged at 10× magnification. Ki67 +ve cells were counted and compared using one‐way ANOVA. Compared to HIKHOM‐1 Gen 1, HIKHOM‐1 Gen 16 had significantly decreased Ki67 +ve cells (4 technical replicates per line, 300–600 cells per replicate, error bars = SEM, *P < 0.05, **P < 0.01, ***P < 0.001, ****P < 0.0001). (D) Fibroblasts analyzed using the wound healing assay. Four technical replicates per genotype were scratched and measured every 2 h for 24 h. Scratch area was analyzed, slope calculated, and the *t* ^1/2^ gap determined (One‐way ANOVA, error bars = SEM, ****P < 0.0001).

We thawed fibroblasts that were obtained from different generations and genotypes and measured proliferation over 7 days (Fig. 3A,B). There were no significant changes in proliferation between HIKHet‐2 Gen1 and HIKHet‐2 Gen6 over 7 days. However, there was a significant decrease in proliferation between HIKHOM‐1 Gen1 and HIKHOM‐1 Gen6 from Day 6 onwards (Fig. 3B). Furthermore, there was a significant decrease in proliferation in HIKHOM‐1 Gen16 compared to both HIKHOM‐1 Gen1 and HIKHOM‐1 Gen6 from Day 4 onwards. It was also noted that HIKHOM‐1 cell lines overall had a higher proliferative rate than HIKHet‐2 cells. This could be attributed to age differences between the donors or possibly due to the homozygous mutation in the *HIKESHI* gene.

To determine the percentage of proliferating cells, cells were fixed and stained for the proliferation marker Ki67 [59] (Fig. S3A). Cell counts indicated that the percentage of Ki67‐expressing cells decreased over generations (Fig. 3C). There was a downward trend between Gen1 and Gen6 of both HIKHet‐2 and HIKHOM‐1 genotypes; however, this decrease was not significant. There was a significant decrease between HIKHOM‐1 Gen1 and HIKHOM‐1 Gen16, suggesting that long‐term culture (over 15 months) slows but does not halt proliferation. Similar to the proliferative assay, the HIKHOM‐1 genotype displayed a higher percentage of Ki67‐positive cells across all generations when compared to the HIKHet‐2 cells.

To analyze the presence of proliferative cells in the biopsy samples after long‐term culture, the biopsies were ultimately fixed, paraffin‐embedded, and immunostained for proliferative markers. Immunofluorescence staining indicates a lack of Ki67 positive and PCNA positive cells in HIKHOM‐1, HIKHet‐2, and unrelated control skin biopsies (data not shown). The validity of this assay was confirmed utilizing multiple antibodies and positive control tissue. Cells were also fixed and stained for the apoptosis marker, Cleaved Caspase 3 (CC3) [60]. Fibroblasts were negative for CC3, indicating minimal, if any, apoptotic cell death (Fig. S3B). Antibody validity was confirmed utilizing iPSCs as positive control cells (Fig. S3B).

To study the proliferative and repair effect of the fibroblasts, the cells were analyzed using a wound healing or scratch assay [48]. There is a significant increase in the *t* ^1/2^ gap of the HIKHOM‐1 Gen16 cells when compared to the other 4 lines (HIKHet‐2 Gen 1, HIKHet‐2 Gen 6, HIKHOM‐1 Gen 1, and HIKHOM‐1 Gen 6), indicating a longer time required to close the gap and lower proliferative capacity (Fig. 3D). There appears to be no significant difference between the *t* ^1/2^ gap rates of the other lines (HIKHet‐2 Gen 1, HIKHet‐2 Gen 6, HIKHOM‐1 Gen 1, and HIKHOM‐1 Gen 6), indicating that the lowered proliferation is linked to the longer‐term culture. The gap area data indicate that the HIKHOM‐1 Gen16 was unable to close the gap after 24 h of culture, whereas the other lines (HIKHet‐2 Gen 1, HIKHet‐2 Gen 6, HIKHOM‐1 Gen 1, and HIKHOM‐1 Gen 6) successfully closed the gap within the 24‐h period (Fig. S4).

### Gene expression and genomic integrity

**Fig. 4:**
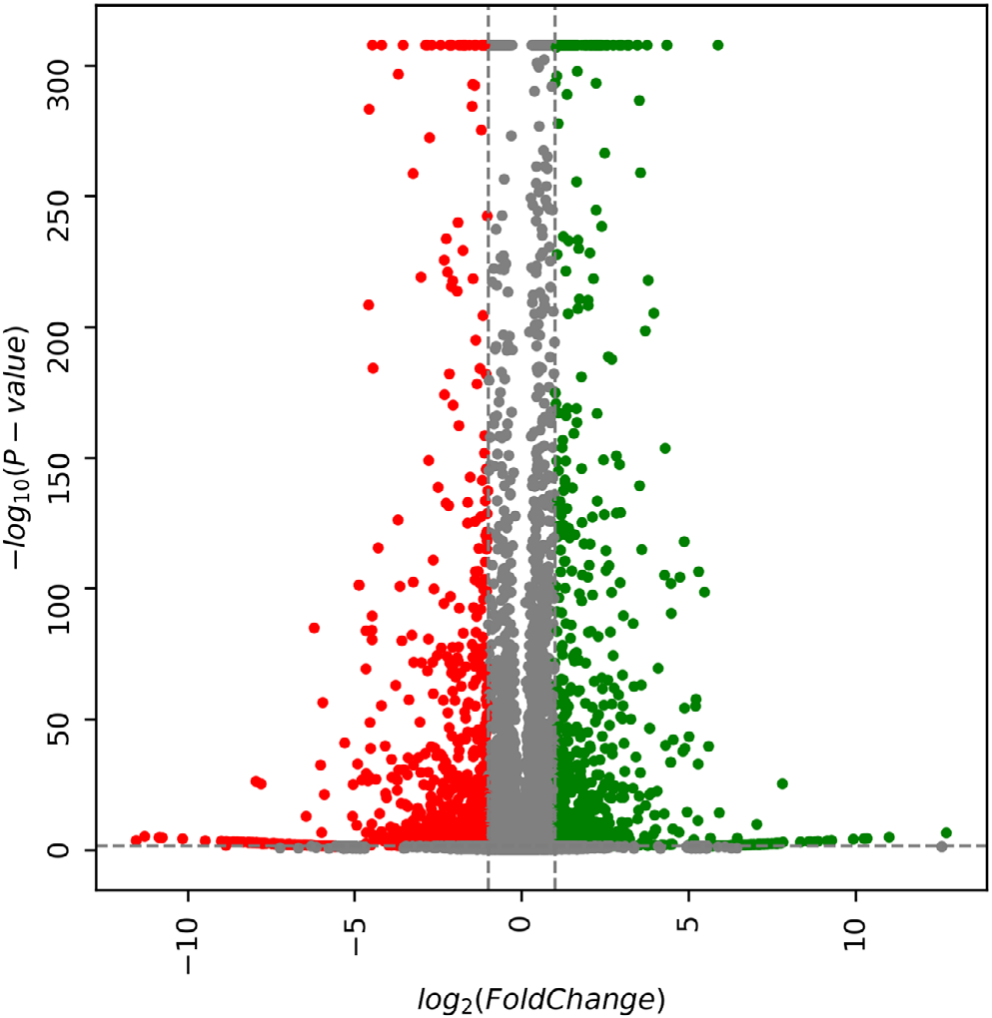
RNA Sequencing shows significant transcriptional changes between HIKHOM‐1 Gen1 and Gen16: RNA was extracted from cell lines and sequenced on the Illumina NovaSeq6000 platform. Data were processed and plotted in a volcano plot with transcripts greater than or less than a 1/−1‐fold change and *P* < 0.001 were deemed biologically and statistically significant.

To determine appreciable differences in gene expression, RNA was obtained from proliferating fibroblast lines from different generations and genotypes. RNA sequencing was conducted, and we observed significant upregulation and downregulation of various genes between HIKHOM‐1 Gen1 and HIKHOM‐1 Gen16 (Fig. 4). Compared to HIKHOM‐1 Gen1, various genes in HIKHOM‐1 Gen16 such as *HAPLN1*, *LHX9*, and *SDK2* were highly upregulated, and *SMO*, *C3*, and *EMILIN2* were highly downregulated (Table S1). Gene enrichment analysis comparing HIKHOM‐1 Gen16 to HIKHOM‐1 Gen1 indicated that a variety of pathways were changed. Pathways relating to the extracellular matrix, ion channel activity, and transmembrane transport were upregulated. Pathways relating to the regulation of the immune system, response to stimulus, and cell surface receptor signaling were downregulated (Table S2). Analysis between HIKHOM‐1 Gen1 and HIKHOM‐1 Gen6 as well as HIKHet‐2 Gen1 and HIKHet‐2 Gen6 also displayed changes in expression and gene enrichment, but substantially fewer changes to expression and pathways were observed, implying long‐term culture amplified transcriptional changes (Fig. S1).

As prolonged time in culture could predispose cells to genomic instability and acquisition of mutations, we processed DNA from proliferating fibroblast lines from different generations and genotypes for low‐pass whole genome sequencing [61, 62, 63, 64]. This allowed us to assess chromosomal stability of long‐term culture and to detect any large DNA sequence gains or losses. Chromosomal maps indicate that there were no significant chromosomal gains or losses that developed over generations (Fig. S2).

Further enrichment analysis determined that between HIKHOM‐1 Gen1 and HIKHOM‐1 Gen16, only a single pathway was significantly changed. A deletion in Chromosome 22 affected genes in the *APOBEC3* cluster including *APOBEC3A*, *APOBEC3B*, *APOBEC3C*, *APOBEC3D*, and *APOBEC3F* (Table S3). This resulted in potential reductions in deaminase activity and negative regulation of viral processes [65, 66, 67, 68]. Despite these genomic changes, the cells displayed no increased susceptibility to contamination. The genomic analysis also indicated that mutations occurred at random and no specific pathway, such as cancer‐related pathways, was targeted.

### Reprogramming competence

**Fig. 5:**
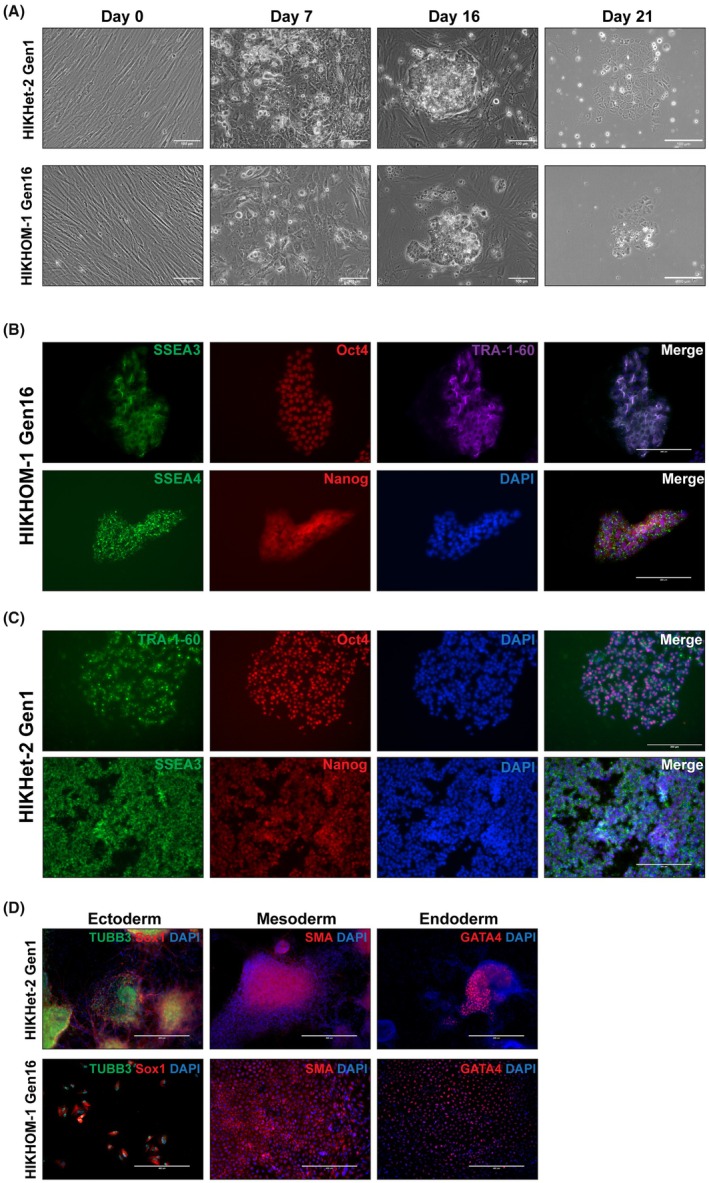
HIKHOM‐1 Gen16 fibroblasts were successfully reprogrammed into iPSCs: (A) Reprogrammed HIKHOM‐1 Gen16 and HIKHet‐2 Gen1 fibroblasts imaged 20 days post‐reprogramming (phase contrast). Cells were observed to change from fibroblast spindle morphology to iPSC appearing colonies. Scale bar: 100 Um. (B) Reprogrammed iPSCs for HIKHOM‐1 Gen16 were fixed and fluorescently stained for pluripotency markers TRA‐1‐60, OCT4, NANOG, SSEA3, and SSEA4. Scale bar: 100 Um. (C) Reprogrammed iPSCs for HIKHet‐2 Gen1 were fixed and fluorescently stained for pluripotency markers TRA‐1‐60, OCT4, NANOG, and SSEA3. Scale bar: 100 Um. (D) HIKHet‐2 Gen1 and HIKHOM‐1 Gen16 iPSCs were grown using trilineage embryoid body assay, fixed, and fluorescently stained for ectoderm (Sox1 and TUBB3), mesoderm (Smooth Muscle Actin) and endoderm (GATA4) markers. Scale bar: 400 Um.

To determine if prolonged propagation of skin biopsies could produce fibroblasts that retain competency for reprogramming to iPSC, HIKHet‐2 Gen1, and HIKHOM‐1 Gen16 fibroblasts were reprogrammed with Sendai virus expressing Yamanaka factors. By Day 16, colonies of round‐shaped cells were evident as well as surrounding cells maintaining a typical fibroblast morphology. On Day 21 after transduction with Sendai viruses, emerging iPSC‐like colonies were manually picked and replated. These were grown out for several more days before being imaged. The individual colonies were flat and consistent with iPSC morphology (Fig. 5A), consisting of small, round cells with a high nuclear/cytoplasmic ratio with prominent nucleoli [69].

To provide further evidence that HIKHOM‐1 G16 fibroblasts retained competency for reprogramming, individual colonies were immunostained for pluripotency markers Nanog, Oct4, TRA‐1‐60, SSEA3, and SSEA4 [70, 71]. All lines stained positive for all markers (Fig. 5B), indicating pluripotent status and successful reprogramming. HIKHet‐2 Gen1 was also reprogrammed and stained for pluripotency as a control (Fig. 5C).

HIKHet‐2 Gen1 and HIKHOM‐1 Gen16 iPSCs were further validated using a trilineage embryoid body assay (Fig. 5D). Both lineages expressed the endoderm marker GATA4, the mesoderm marker Smooth Muscle Actin, and the ectoderm markers Sox1 and β‐III‐Tubulin, indicating that both cell lines can be successfully differentiated into the three germline lineages [72, 73].

## Discussion

This study demonstrates the long‐term viability and reprogramming competency of skin biopsy‐derived fibroblast cultures. An almost 16‐month culture of a single 3 mm skin biopsy produced 16 generations of fibroblasts amounting to many millions of cells. These fibroblasts were validated for a variety of experiments including immunofluorescence, proliferation, RNA and DNA sequencing, and iPSC reprogramming. We observed retention of fibroblast morphology and marker expression, further supporting the long‐term maintenance of cellular identity.

Proliferation assays showed that later generations have decreased proliferative capacity compared to the first generation of fibroblasts. However, the maintenance of proliferation demonstrates the potential for clinical usage to produce large quantities of cells. HIKHOM‐1 fibroblasts did show an overall higher level of proliferation compared to HIKHet‐2 cells, which could be due to genotype as well as age differences [74]. At the end of the prolonged culture period, we did not observe proliferative cells in the fixed and sectioned skin biopsies. This possibly may be an artifact of our system, as it has been reported that extended formalin fixation can reduce Ki67 staining [75]. Wound healing assay indicated similar decreases in proliferation in higher‐generation fibroblasts compared to lower‐generation fibroblasts. Cultured fibroblasts were negative for Cleaved Caspase 3 (CC3) cells, indicating minimal, if any, apoptotic cell death. As newly plated fibroblasts were not confluent, the fibroblasts appear to be continuously proliferative but exhibited contact inhibition [76, 77]. As cultured fibroblasts were passaged or frozen upon confluency, this could result in a lack of apoptotic pressure on the cells.

RNA and DNA sequencing demonstrated overall maintenance of genomic integrity over long‐term culture. There were no large DNA/chromosomal abnormalities over fibroblast generations. The few acquired mutations seem to have occurred at random and did not appear to specifically target a pathway or affect the physiology and culture potential of the cells. Studies on other cell types such as mesenchymal stem cells have demonstrated that long‐term culture can result in random mutation accumulation, with oxidative stress of culture conditions playing a primary role [62, 78, 79]. Further experiments may address if regulation of culture conditions to reduce oxidative stress and other stressors will reduce mutation frequency in long‐term skin‐biopsy culture.

Extensive changes in expression were observed between the first and last generation, similar to studies observing long‐term culture of mesenchymal stem cells [80]. The resulting protein changes and functional differences caused by these expression changes will require further study. Successful reprogramming of Gen16 fibroblasts indicated that reprogramming competence was not altered over long‐term fibroblast culture. If long‐term fibroblast culture is utilized in clinical applications, both for fibroblast therapy and for reprogramming, cells should be further sequenced to ensure that no harmful mutations have been acquired that may reduce therapeutic potential, lead to tumorigenesis, or cause a host response.

In summary, we demonstrate the capacity and robustness of skin biopsies to continuously produce many generations of primary fibroblasts. With a variety of applications in burn and wound healing therapeutics, as well as stem cell reprogramming and rare disease study, these findings have important implications for primary fibroblast culture and their use for translational research.

## Conflict of interest

The authors declare no conflict of interest.

## Author contributions

SR‐K and KCE conceived and designed the study. SR‐K, KCE, and GMW performed experiments as well as data collection. SR‐K, MAR, and KCE analyzed the data and interpreted the results. SR‐K and KCE wrote the initial manuscript draft. All authors reviewed the results and approved the final version of the manuscript.

## Supporting information


**Fig. S1.** RNA Sequencing shows significant transcriptional changes.
**Fig. S2.** DNA Sequencing shows lack of chromosomal changes.
**Fig. S3.** Immunofluorescence for proliferative and apoptotic markers across cell lines.
**Fig. S4.** Gap closure over time in wound healing assay to measure cellular proliferation.

**Table S1.** Analysis of RNA sequencing data.

**Table S2.** Gene enrichment analysis of RNA sequencing data.

**Table S3.** Gene enrichment analysis of DNA sequencing data.

## Data Availability

RNA Sequencing data was deposited in Gene Expression Omnibus (GEO) under accession GSE271347.
