# Supplementary material for: Long‐term culture of skin biopsies: maintenance of fibroblast production and competency of reprogramming
Source: FEBS Open Bio. 2025 Dec 19;16(2):397–411. doi: 10.1002/2211-5463.70136 (PMC12871563; doi:10.1002/2211-5463.70136)
Supplement: Supplementary file 1 — Fig. S1. RNA Sequencing shows significant transcriptional changes. Fig. S2. DNA Sequencing shows lack of chromosomal changes. Fig. S3. Immunofluorescence for proliferative and apoptotic markers across cell lines. Fig. S4. Gap closure over time in wound healing assay to measure cellular proliferation. [file FEB4-16-397-s003.docx]

**SUPPLEMENTARY FIGURES**


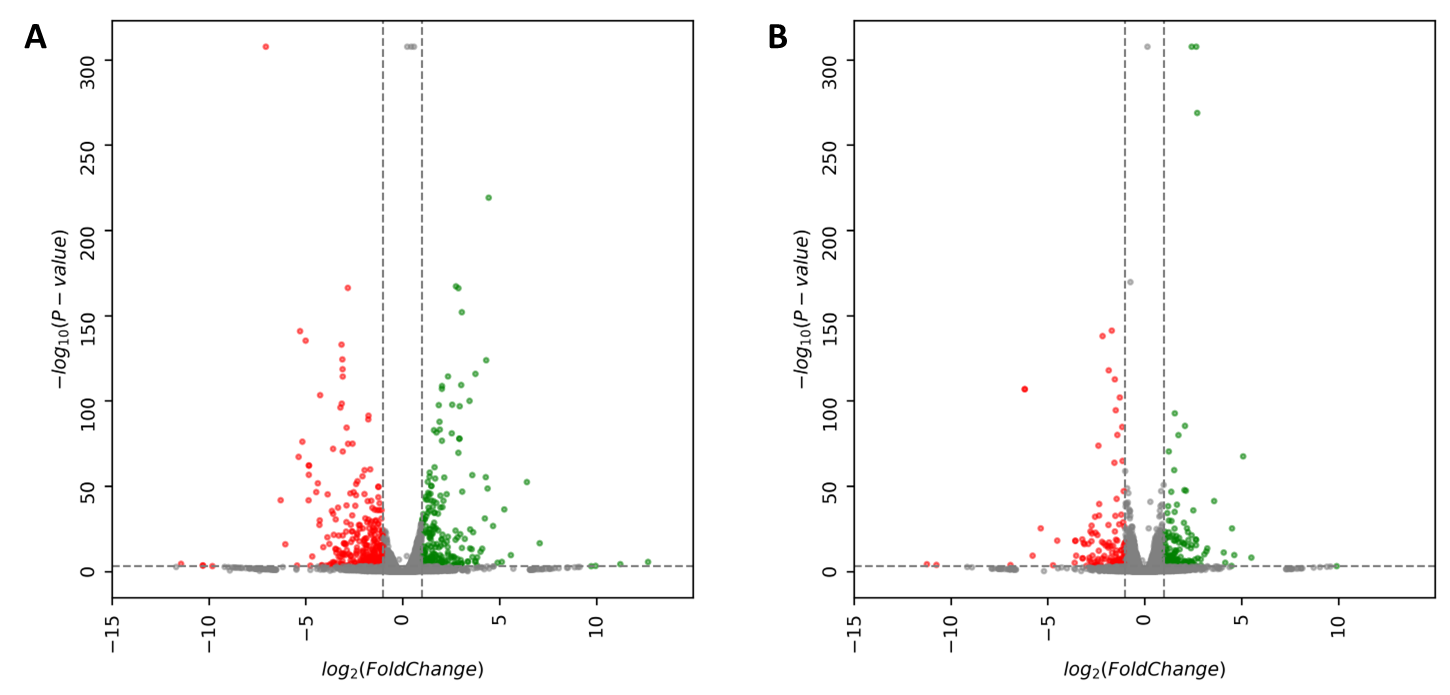


**Supplementary Figure 1. RNA Sequencing shows transcriptional changes.** RNA was extracted from cell lines and sequenced on the Illumina NovaSeq6000 platform. Data was processed and plotted in a volcano plot with transcripts greater than or less than a 1/-1-fold change and p<0.001 were deemed biologically and statistically significant. (A) Changes observed between HIKHOM-1 Gen1 and Gen6 (B) Changes observed between HIKHet-2 Gen1 and Gen6.


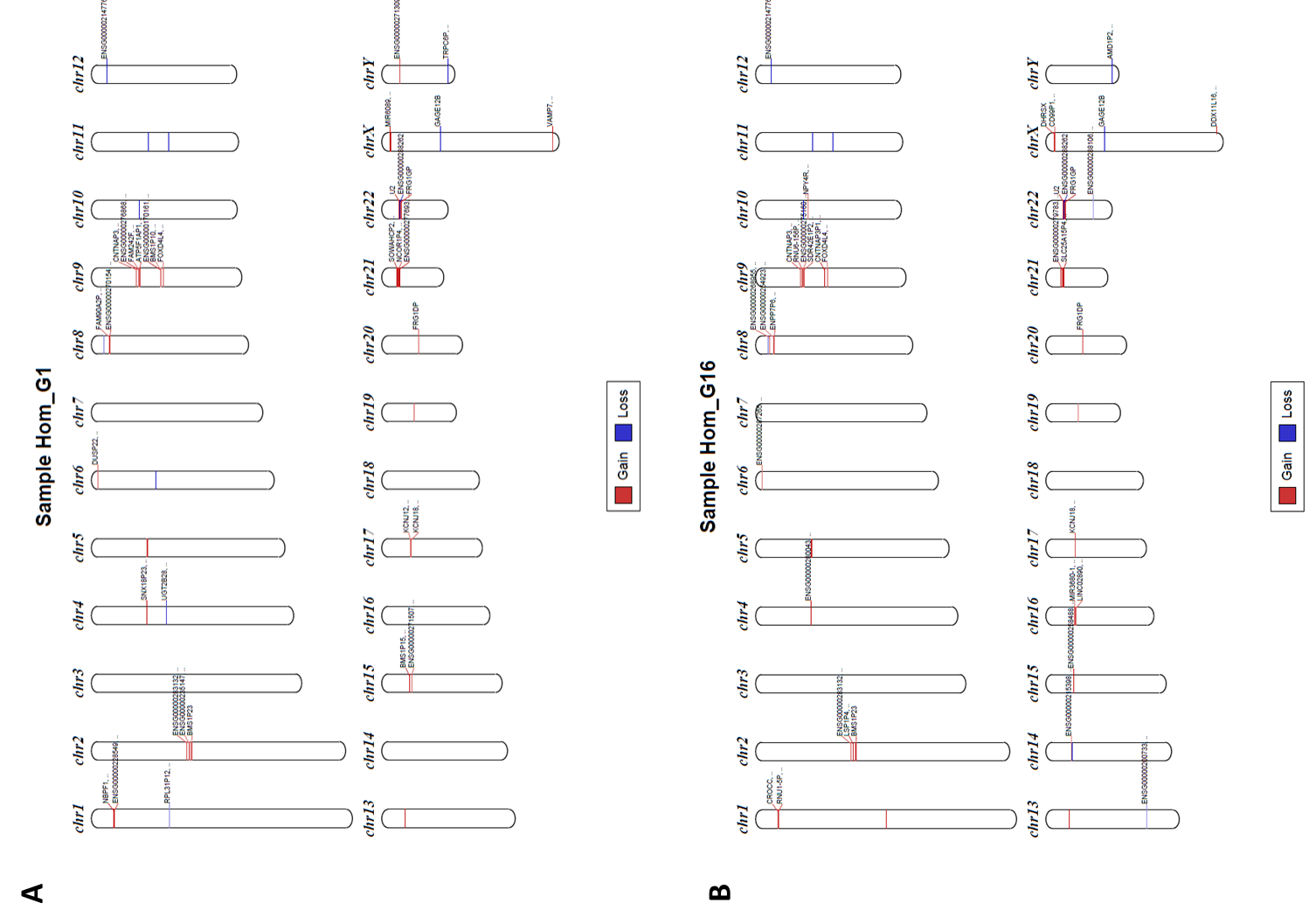


**Supplementary Figure 2. DNA Sequencing shows lack of chromosomal changes.** Sample were analyzed using low-pass whole genome sequencing which indicates lack of large DNA gains or losses.

**
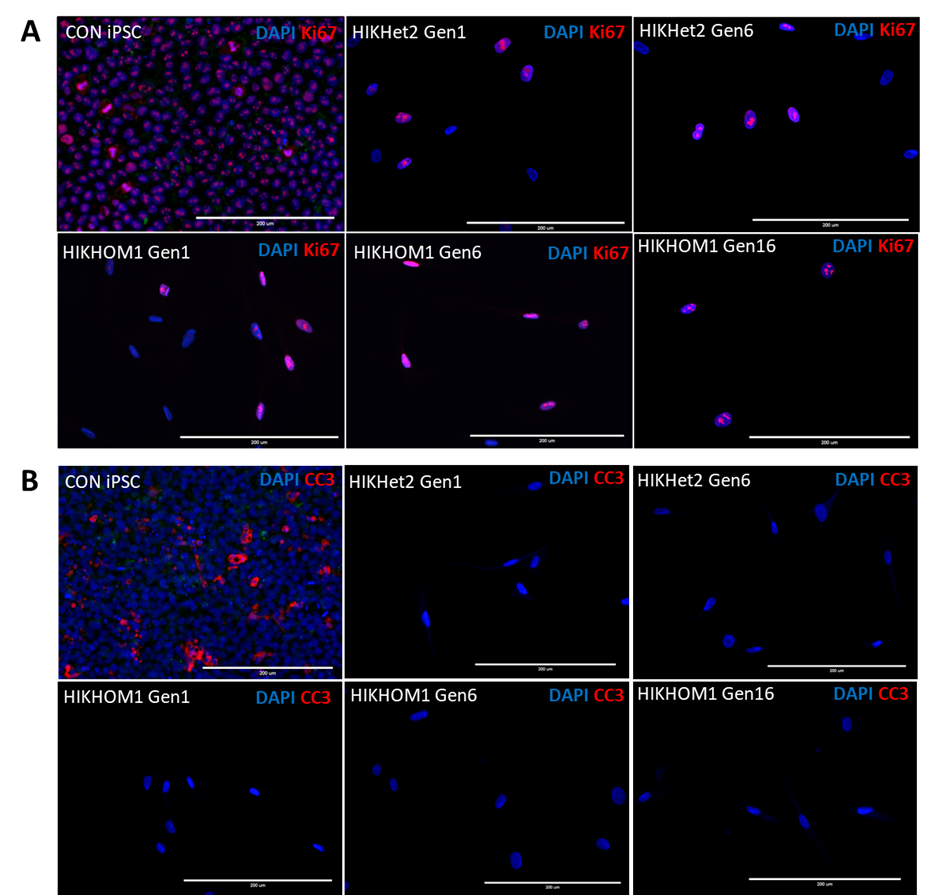
**

**Supplementary Figure 3. Immunofluorescence for proliferative and apoptotic markers across cell lines.** (A) Cell lines were fixed and stained for proliferative marker Ki67 which tested positive in all lines. Unrelated iPSC cells are used for positive control. Scale bar: 200 um. (B) Cell lines were fixed and stained for Cleaved Caspase 3 and were negative for apoptosis. Unrelated iPSC cells are used for positive control. Scale bar: 200 um.


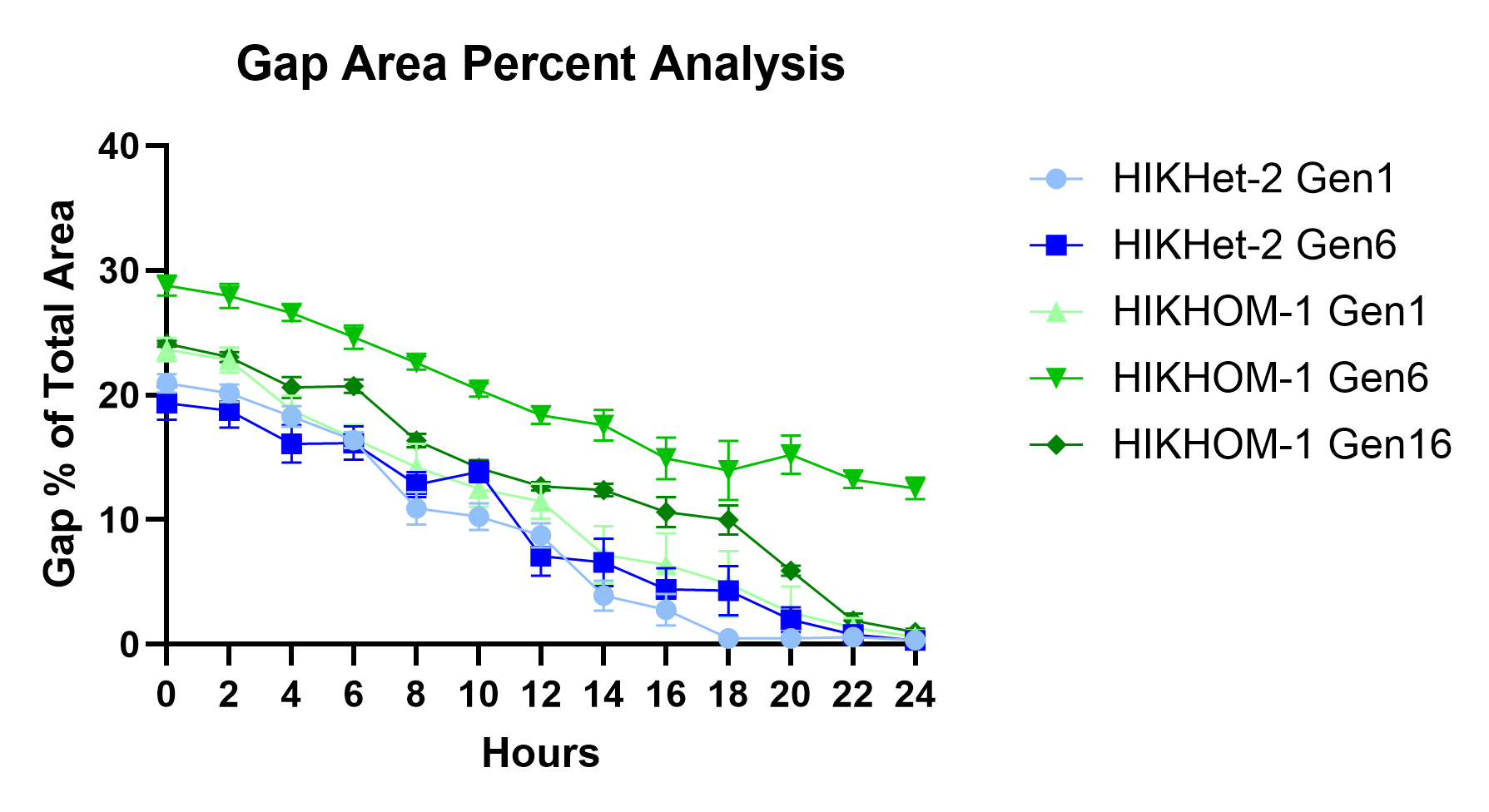
**Supplementary Figure 4. Gap closure over time in wound healing assay to measure cellular proliferation.** Fibroblasts were plated and scratched. Scratch gap was measured every 2 hours for 24 hours at 4x magnification. Gap area was calculated using ImageJ and plotted as decrease in gap area over time (n=4 wells per cell line, error bars = SEM).
